# Risperidone Induced Hyperprolactinemia: From Basic to Clinical Studies

**DOI:** 10.3389/fpsyt.2022.874705

**Published:** 2022-05-06

**Authors:** Milena Stojkovic, Branimir Radmanovic, Mirjana Jovanovic, Vladimir Janjic, Nemanja Muric, Dragana Ignjatovic Ristic

**Affiliations:** ^1^Department of Psychiatry, Faculty of Medical Sciences, University of Kragujevac, Kragujevac, Serbia; ^2^Psychiatric Clinic, University Clinical Center Kragujevac, Kragujevac, Serbia

**Keywords:** Risperidone, antipsychotics, prolactin, hyperprolactinemia, side-effects, basic studies, clinical studies

## Abstract

Risperidone is one of the most commonly used antipsychotics (AP), due to its safety and efficacy in reducing psychotic symptoms. Despite the favorable side effect profile, the therapy is accompanied by side effects due to the non-selectivity of this medicine. This review will briefly highlight the most important basic and clinical findings in this area, consider the clinical effects of AP-induced hyperprolactinemia (HPL), and suggest different approaches to the treatment.The route of application of this drug primarily affects the daily variation and the total concentration of drug levels in the blood, which consequently affects the appearance of side effects, either worsening or even reducing them. Our attention has been drawn to HPL, a frequent but neglected adverse effect observed in cases treated with Risperidone and its secondary manifestations. An increase in prolactin levels above the reference values result in impairment of other somatic functions (lactation, irregular menses, fertility) as well as a significant reduction in quality of life. It has been frequently shown that the side effects of the Risperidone are the most common cause of non-compliance with therapy, resulting in worsening of psychiatric symptoms and hospitalization. However, the mechanism of Risperidone-induced HPL is complicated and still far from fully understood. Most of the preclinical and clinical studies described in this study show that hyperprolactinemia is one of the most common if not the leading side effect of Risperidone therefore to improve the quality of life of these patients, clinicians must recognize and treat HPL associated with the use of these drugs.

## Introduction

Hyperprolactinemia is one of the most common side effects of Risperidone treatment. Previous studies report that the frequency of this side effect is >30% (1, 2). If we take into account that Risperidone is one of the most commonly prescribed antipsychotics, this side effect becomes even more significant (3, 4). In the current era of de-institutionalization of patients with serious mental illness with more attention paid to better social engagement and inclusion in work and social activities, numerous studies have shown a higher incidence of poor physical health in patients with mental illness (5, 6). Because mental illness affects many aspects of patient's lives, social relations, and daily functioning, the unintended side-effects of Risperidone, such as HPL, may worsen patient's social relationships and interactions and contribute to physical disability. Those effects of Risperidone therapy are of prime clinical importance and must be considered in the management of patients.

## Pharmacodynamics of Risperidone

Although Risperidone was the second atypical AP developed after clozapine, it quickly became the first line of treatment for schizophrenic patients. The pharmacological development of this drug is based on its precursor called ritanserin, which is a 5-HT2A serotonin receptor blocker. By chemical modification, a compound with a predominant pharmacological effect of D2- / 5-HT2A receptor antagonism was synthesized, which had a stronger effect on 5-HT2A than D2 receptors. This pharmacological profile was later the basis for the development of other serotonin and dopamine antagonists. Risperidone development was an important step forward toward new treatment options for psychotic patients (4).

This drug, an antagonist of the D1 (D1 and D5) and D2 (D2, D3, D4) family of receptors, blocks the mesolimbic pathway, the limbic pathway of the prefrontal cortex, and the tuberoinfundibular pathway in the central nervous system. On the other hand, it shows activity at alpha α1 and α2 adrenergic receptors, H1 histamine receptors, and serotonin receptors (7–9) with a stronger effect on 5-HT2A compared to D2 receptors (4). The efficacy of this drug has been proven in many clinical conditions, such as schizophrenia, bipolar I acute manic or mixed episodes as monotherapy (in adults and children aged 10 and up), bipolar I acute manic or mixed episodes adjunctive with lithium or valproate (in adults), and autism spectrum disorders (10–13). Patients with schizophrenia may exhibit a range of receptor abnormalities, such as abnormalities in D1, D2, serotonin, α1 and α2, muscarinic cholinergic, histamines, GABA, sigma opioid receptors, and glutamate systems (NDA). These abnormalities can lead to inadequate prolactin secretion, which is further affected by psychotropic medication (13–15). Knowing that the key role in the development of a manic episode is predominantly dopaminergic signaling, while the opposite underlies depression, it is clear why Risperidone is an effective treatment for bipolar I disorder (16). Autism spectrum disorder (ASD) appears during early childhood and is associated with genetic abnormality in more than 30% of patients and environmental factors. Lack of a clear biochemical basis for its occurrence and has been a challenge for clinicians to treat (17). Risperidone is one of two drugs approved by the U.S. Food and Drug Administration (FDA) for the treatment of ASD (18).

## Prolactin, Dopamine, Hyperprolactinemia

**Figure 1:**
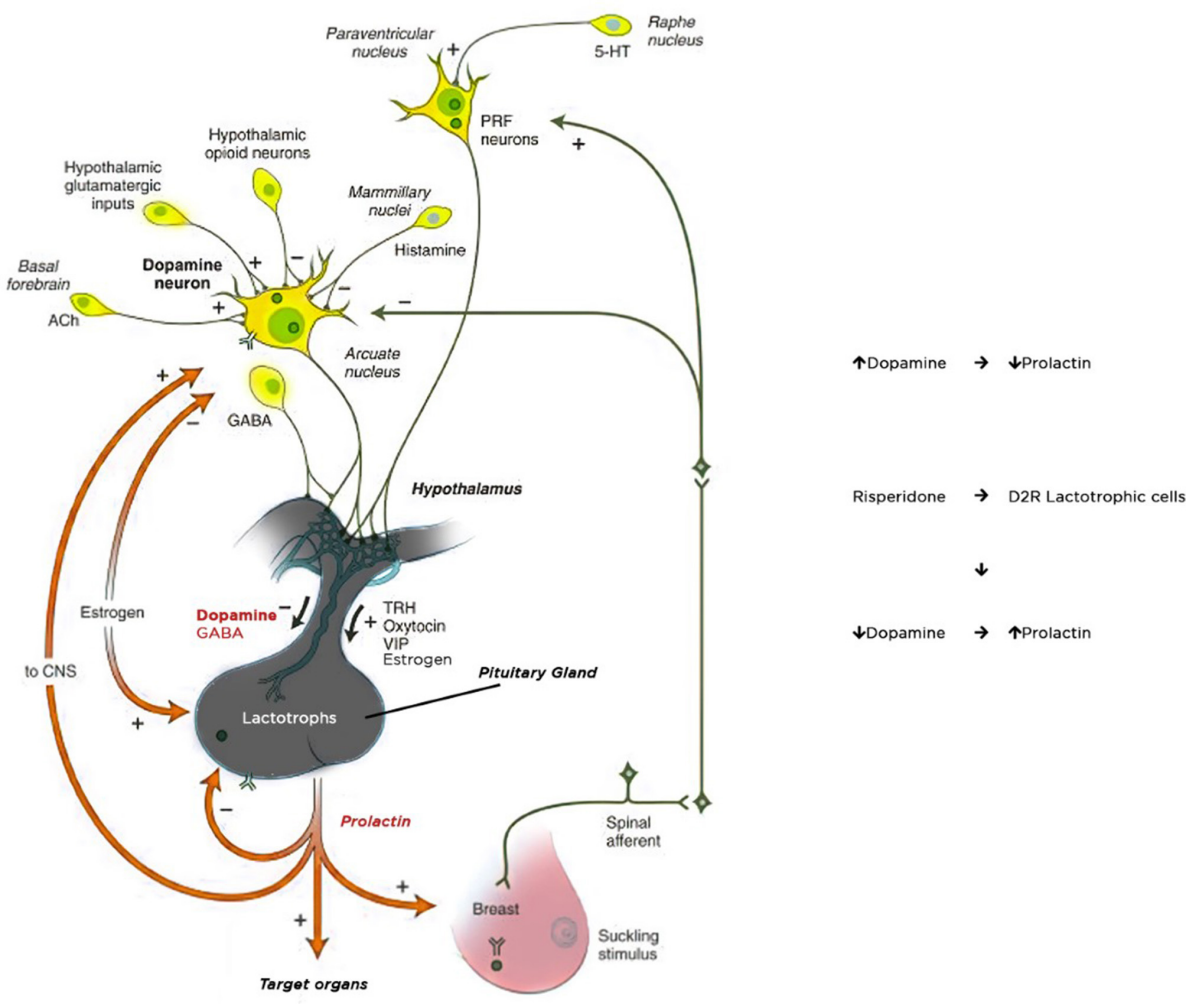
Mechanism of prolactin secretion and hyperprolactinemia.

The main physiological control of prolactin secretion is performed by the inhibitory effect of dopamine. Dopamine is synthesized and released in the hypothalamic periventricular zone as a result of activity in the periventricular and arcuate nucleus. It reaches the anterior pituitary gland through portal vessels and inhibits prolactin secretion by binding to D2 receptors located on the surface of the pituitary lactotroph cells. Dopamine acts as a PRL-inhibiting factor on D2 receptors, whereas serotonin stimulates prolactin secretion (19). Dopamine receptors achieve their effects through various mechanisms, such as activation/inhibition of adenylyl cyclase, modulation of Ca^++^ and K^+^ channel activity, Na^+^/H^+^ exchange, and Na/K-ATPase activity, alterations in arachidonic acid metabolism as well as by influencing signaling pathways involved in cellular mitogenesis and differentiation (20, 21). Prolactin release is stimulated by strong nipple stimulation during breastfeeding and may be released in response to physical and emotional stress. Some CNS hormones including TRH, oxytocin, vasopressin increase prolactin secretion, while major neurotransmitters, gamma-aminobutyric acid (GABA) and acetylcholine, inhibit prolactin secretion (22) (Figure 1).

HPL is defined as an increase in the concentration of prolactin levels beyond normal values which vary depending on the assay technique used. The maximum cut-off values range from 15 to 25 g / L in women and 15 to 20 g / L in men (23, 24). Increased prolactin secretion may occur as a primary disorder of autonomic pituitary prolactin tumors–prolactinoma. Much more common is secondary HPL due to a lack of hypothalamic inhibition by prolactin inhibition factor (PIH). Certain drugs may affect the main regulatory prolactin mechanisms, causing its levels to increase or decrease (25). Among the medications which cause HPL, psychotropic drugs with an effect on dopamine antagonism, play an important role (26).

The major symptoms of HPL lead to impairment of other somatic functions. The symptoms of chronic HPL include reproductive dysfunction (anovulation, menstrual irregularity, all the way to amenorrhea, decreased fertility, decreased estrogen and testosterone production), sexual impairment (decreased libido, retrograde or painful ejaculation, erectile dysfunction orgasmic dysfunction, impotence), breast pathology (galactorrhoea, breast enlargement, prolactin-sensitive dysplasia with increased potential for breast cancer, gynecomastia), abnormalities associated with chronic hypogonadism (decreased bone mineral density and osteoporosis, increased cardiovascular risk, the appearance of metabolic syndrome, development of certain tumors), behavioral and mood alterations (depression, anxiety, hostility, memory deficit, psychosis) (3, 27, 28). Altogether, these effects may lead to infertility, which is an important health concern. The effects on the reproductive system occur both as a direct action of prolactin on target tissues (ovaries and testes) and as indirect effects mediated by the decrease in pulsatile gonadotropin secretion with a resulting decrease in FSH and LH secretion and leading to gonadal dysfunction (28) (Table 1).

**Table 1:** Manifestations and the consequences of hyperprolactinemia.

**Short-term side effects**	**Long-term side effects**
•Menstrual disorders •Anovulation •Galactorrhea •Breast enlargement •prolactin-sensitive dysplasia •Gynecomastia •decreased fertility •decreased estrogen and testosterone production •Impotence in men •Decreased libido •retrograde or painful ejaculation •erectile dysfunction •orgasmic dysfunction •Syndrome: Amenorrhea - Galactorrhea Azoospermia - Gynecomastia	•Osteoporosis, •Breast cancer •Development of certain tumors, Prolactinoma, •Metabolic syndrome •Erectile dysfunction •increased cardiovascular risk •behavioral and mood alterations •depression, •anxiety, •hostility, •memory deficit, •psychosis

## Basic Studies

The importance of preclinical research is reflected in identifying and determining the dangers of acute or chronic drug use. In recent decades, the range of indications for this drug has expanded and the number of adults and children prescribed APs for the treatment of psychiatric conditions has increased (13). According to Kapur et al. (29) atypical APs with a tendency to increase prolactin show higher pituitary binding relative to the striatal D2 receptor. The degree of increase in prolactin levels is reflected in the difference between central and peripheral binding of D2 receptors, as well as that prolactin levels are dose-dependent with all APs. Moreover, they showed that higher peripheral potency of drugs leads to a greater increase in prolactin levels, and different passage through the blood-brain barrier affects prolactin levels (29). On the other hand, Rourke et al. (30) found that prolactin levels in rats on atypical AP treatment may not be fully predictable for clinical practice. According to Rourke, Risperidone administration had similar effects on prolactin levels in rats after acute and chronic administration, causing a statistically significant increase in prolactin levels, which were present 24 h after dosing following acute administration. The temporal nature of the response to prolactin over 24 h varies. The magnitude of the increase in prolactin after Risperidone treatment on day 7 compared to day 1 and 28 was smaller, the data also showed that Risperidone levels were three times higher in the plasma compared to the level in brain tissue on day 7, compared to similar levels on days 1 and 28 (30).

Assuming that the action of Risperidone in pituitary lactotrophs is similar to GH3 cells, derived from a pituitary tumor from a female rat, Wu et al. (31) demonstrated that Risperidone may affect membrane excitability and prolactin secretion in GH3 cells. They investigated the electrophysiological effects of Risperidone in the GH3 clone cell line and found that susceptibility to RIS in pituitary lactotrophs depends on pre-existing resting membrane potential levels, action potential activation rate, or concentration of Risperidone used. The results indicate that inhibition in GH3 cells is not associated with dopamine receptor blockade and would significantly contribute to the change in membrane potential, thus affecting prolactin secretion (31). Continuing the claim that high prolactin levels are associated with potentially adverse effects on the reproductive system, including neuroendocrine effects through inhibition of GnRH secretion. Görmüş et al. (32) discovered important markers of reproductive toxicity by histopathological examination of the testes, demonstrating that hormone levels that play an important role in spermatogenesis, sperm quality parameters, and biomarkers of oxidative stress. In addition to HPL, Risperidone also causes damage to the hypothalamic-pituitary-gonadal axis. The results of the study showed that RIS caused a decrease in FSH and testosterone levels, testicular damage, and selectively destroyed Leydig cells. Histopathologically, it worsens sperm morphology and concentration and plays a role in the development of oxidative stress by reducing dose-dependent GSH levels and increasing malondialdehyde (MDA) levels, without significantly affecting superoxide dismutase (SOD) and catalase (CAT) (32). Furthermore, Elmorsy et al. (33), had comprehensive *in vitro* effects of APs, including Risperidone, on the ovaries of female rats. They show a significant increase in reactive oxygen species (ROS) in interstitial cells. The most significant effect of APs is in the mechanism of lipid peroxidation and a significant increase in the production of thiobarbituric acid reactive substances (TBARS). In terms of antioxidant defense, intracellular total glutathione is significantly reduced, as is the activity of all antioxidant protection enzymes in human serum. This is supported by the fact that supplementation with antioxidants (reduced GSH or quercetin) significantly reduces the cytotoxic effect (33).

## Clinical Studies

The incidence of HPL in patients receiving APs varies between studies but can reach 70–91% in both sexes, with a lower incidence in men (5). Compared with olanzapine, Risperidone, and atypical APs are more likely to cause this side effect (34). HPL is one of the most common side effects of Risperidone, with a frequency of more than 30% (35). The use of PET confirmed the significance of differences in the concentration of APs between the brain and plasma (B / P ratio). The B / P ratio is lower for Risperidone than for olanzapine and haloperidol (36). Risperidone leads to a higher increase in prolactin levels than other atypical APs due to an incomplete crossing of the blood-brain barrier. The passage through the blood-brain barrier is delayed due to the smaller lipophilic profile, so it stays longer in the tuberoinfundibular pathway, where the anterior pituitary gland is located. Due to the longer presence outside the blood-brain barrier, D2 receptor occupancy is higher in the pituitary gland than in the striatum (37). Higher doses of the active form of the drug in plasma are generally associated with greater blockade of the D2 receptors. Blockade leads to loss of inhibitory factor dopaminergic prolactin in lactotrophic cells. Which explains the fact that APs with a higher D2 binding index produce a larger and more frequent increase in prolactin (38, 39).

Moreover, Risperidone is administered in different forms (tablets, solution, long-acting injections), and that the method of application affects the level of the drug in the blood, such as daily variations, but also the total concentration (40, 41). HPL is recognized in more than 72% of the females treated with oral Risperidone and 53% treated with long-acting intramuscular injection of Risperidone (LAIR) (42, 43). It has been shown that the replacement of conventional depot APs with LAIR can lead to higher serum prolactin levels, up to 55.85 ng/mL, as well an increase in the prevalence of HPL to 75% for men and 91.7% for women vs. 38.8 and 58.3%, respectively (42). Compared with conventional drugs, it has been found in several different datasets that prolactin levels raised by LAIR were higher and more prolonged than those induced by haloperidol (24, 43). A higher prolactin-releasing capacity of Risperidone cannot be attributed to its ability to block serotonin receptors, since serotonin has a positive influence on prolactin secretion (44). Similarly, it has been demonstrated that the maximal D2 binding was achieved by treatment with either LAI haloperidol or Risperidone, while baseline prolactin levels increased significantly during Risperidone treatment (from 35.0 ± 16.0 to 55.7 ± 19.6 ng/ml) and maximal prolactin levels were beyond those achieved with high doses of haloperidol (45). Although is a significant increase in prolactin levels in both sexes, followed by significant gender differences (females were more subjected to higher prolactin levels), the changes in serum prolactin levels were not associated with the reduction of psychopathology (46, 47).

In contrast to other atypical APs, Risperidone therapy is followed by a significant, dose-dependent elevation of prolactin levels. On the other hand, clozapine, olanzapine, and quetiapine are associated with decreases in prolactin (48–51). Compared to olanzapine, HPL was more common in Risperidone-treated patients (51.73 vs. 8.23 ng/mL; *p* < 0.001), followed by a sexual dysfunction (49). In contrast to aripiprazole, RIS produced a significantly greater increase in mean prolactin levels. The percentage of patients with an increase in serum prolactin level above the upper limit of the reference range in each group was 90.5% vs. a decrease from prolactin baseline levels in aripiprazole treatment groups (52). Moreover, Feng et al. (53), showed a significant increase in glucose levels and body mass index for Risperidone.

Due to the expanding range of indications for this drug, Risperidone is increasingly used in pediatric clinical practice for the treatment of schizophrenia, bipolar I acute manic or mixed episodes as monotherapy, and autism-associated irritability (54). Given that side effects, especially HPL, have been shown to predispose to growth and puberty disorders in children, the importance of measuring prolactin levels and preventing, and monitoring the development of side effects, as well as their adequate treatment in this population, was particularly emphasized (55, 56). An increase in prolactin levels in this age is sex-dependent and depends on the type of AP and the duration of psychosis (55–57).

Many studies investigated the frequency and association between Risperidone and prolactin-related symptoms (41, 58–64). The frequency of endocrinologic side effects is around 1 to 2% in patients taking Risperidone (58). A good correlation existed between sex and age at the onset. Females and younger patients are more sensitive to elevated prolactin levels, with the possibility for persistent HPRL a long time after acute treatment (59–61). A positive association of female gender and RIS dose with PRS severity was also confirmed. Patients on chronic therapy may exhibit a macroprolactinoma (60). Moreover, a reduction in the severity of prolactin-related symptoms was demonstrated during 1 year of treatment, as well as a reduction in the prevalence of prolactin-related symptoms, which can be attributed to drug tolerability as well as drug withdrawal (62). Although the side effect profile of Risperidone appears to correspond somewhat to serum levels, it has been difficult to demonstrate a positive association between serum prolactin levels and the clinical efficacy of Risperidone according to the medical literature. Zhang et al. (63) found a relationship between the improvement in positive symptoms and the change in serum prolactin level. In contrast to this, Lee et al. (46) showed no significant correlation of changes in serum prolactin with the clinical efficacy of Risperidone.

All the above basic and clinical findings are listed in Table 2.

**Table 2:** Main findings related to basic and clinical studies.

**Study design**	**No of the subjects** **include / RIS**	**Study population**	**Duration of treatment**	**RIS dosage (BS: mg/kg** **BW CS: mg/day)**	**PRL (ng/ml)**	**PRS**	**References**
BS	77 / 20	M-Wiga Wistar rats F-CD rats	1 day	0,0025	6.6 ± 3.2	/	(29)
				0.01	^*^50 ± 7		
				0.04	^*^469 ± 149		
				0.16	^*^560 ± 132		
				^*^PRL↑ DD			
BS	48 / 24	M-CD rats	1 day	0.01	20	/	(30)
				0.032	44 ± 1^*^		
				0.1	48 ± 1^*^		
				0.32	42 ± 1^*^		
				^*^PRL ↑ DD			
	12 / 6		28 days (1,7,28)	0.32	^*^PRL ↑ days 1, 7, 28		
OL	65 / 21	SCH SAD SCHD	54 weeks	4–10 mg/day	bPRL: 27.2 ± 45.5 etPRL:^*^107.0 ± 71.1	/	(34)
	339 / 167		28 weeks	4–12 mg/day	bPRL: 26.1 ± 34.9 etPRL: ^*^71.5 ± 59.0		
RS	422 / 56	PD SCH SAD MD	/	RIS-CPZ equivalents	P (%) HPRL, 91%; 64% having a 2-fold increase	Amenorrhea, galactorrhea, inhibition of ejaculation, erection disturbance, breast sensitivity	(35)
	422 / 24			CA + RIS (CPZ equivalents)	P HPRL 100%; 63% having a 2-fold increase		
OL	24/24	SCH	12 weeks	LAIR M 30.7 ± 6.4 mg/2 weeks	^*^PRL ↑ bPRL: 27.67 ± 28.5 etPRL: ^*^55.85 ± 43.0 P (%) HPRL ↑ M 33.3% → 75% F 58.3% → 91.7%	dysmenorrhea	(42)
OL	12	SCH	6 weeks	8–16 mg/day	^*^PRL ↑ bPRL:35.0 ± 16.0 etPRL: ^*^55.7 ± 19.6	/	(45)
OL	27	SCH, SAD	12 weeks	2–4 mg/day	^*^PRL ↑ bPRL:0 2 week: 90.0 ± 57.7 4 week:102.6 ± 70.8 8 week 83.8 ± 47.8 12 week:83.0 ± 48.6 ^*^PRL↑ correlated with doze ^*^↑PRL F > M	menstrual abnormalities galactorrhea erectile dysfunction	(46)
DB, R	555/278	SCH SAD SCHD	3 months	4–8 mg/day	P (%) HPRL 73.8% F: mPRL 73.69, M mPRL = 34.08	gynecomastia, galactorrhea	(47)
R, DB	329/ 164	BD	3 weeks	1–6 mg/day	^*^PRL ↑ 51.73	sexual dysfunction	(49)
NAT	50 / 22	B, SCH, SAD ADHD, A., AD, DO, MD	≥6 months	Mean 1,5 mg/day	^*^mPRL↑ (22.0 ± 1.9).	menstrual or breast problems	(50)
R, DB	308 / 152	SCH SAD	6 weeks	4–6 mg/day	bPRL: 22.3 ± 27.6 etPRL: 62,6 ± 30 PRL↓-10.1 ± 3.6 ng/ml with quetiapine	/	(51)
NAT, OL, R	555 / 46	SCH	26 weeks	2–6 mg/day	^*^PRL↑ bPRL:0 etPRL: 51.3 mg/dl	sexual dysfunction	(52)
R, DB	194	SCH	6 weeks	2–6 mg/day	^*^PRL↑ 60.4 vs. lurasidone 3.5		(53)
P	40 / 11	SCH, PD, MD,DO, PDD, IED, ED	12 weeks	2 mg/day	P(%) HPRL 71% bPRL: 25.3 etPRL: 46.8	breast tenderness, irregular menses, decreased libido, erectile dysfunction, galactorrhea, amenorrhea	(55)
R	170	SCH, PDD, SAD, PD, BD	>6 months	5 mg/day	P(%) HPRL70.6% F: 66.7%; M: 33.3% ^*^PRL↑ F > M (2.46xNV> 1.59xNV) F, AP, DoP >10 years ^*^↓PRL > 10 years DoT	/	(57)
OL	20	SCH SAD	0,8 – 20 years	2–8 mg/day	P HPRL 65% ^*^F, younger age	Amenorrhea, menstrual dysfunction	(61)
OL	30	SCH	12 weeks	6 mg/day	^*^bPRL SCH↓ bPRL SCH: 4.5 ± 3.2 ↔ bPRL healthy controls 9.6 ± 4.9 ^*^etPRL↑ SCH: 27.2 ± 15.3	/	(63)

*RIS, risperidone; PRL, prolactin; PRS, prolactin related symptoms; BS, basic study; BW, body weight; M, male; F, female; CD, Sprague Dawley; DD, dose depending; OL, open-label design; SCH, schizophrenia; SAD, schizoaffective disorder; SCHD, Schizophreniform disorder; bPRL, baseline prolactin; etPRL, end-time prolactin; RS, retrospective study; PD, psychotic disorder; MD, mood disorder; CPZ, chlorpromazine; CA, a conventional antipsychotic; P, prevalence; HPRL, hyperprolactinemia; LAIR, long-acting risperidone; DB, double-blind; R, randomized; NAT, naturalistic; BD, bipolar disorder; ADHD, attention-deficit/hyperactivity disorder; AS, Asperger syndrome; A, autism; DO, Disruptive disorder; IED, intermittent explosive disorder; PDD, pervasive developmental disorder; ED, eating disorder; NV, normal values; DoP, duration of psychosis; DoT, duration of treatment; AP, antipsychotic medication; P, prospective study.*

* *- significant result; ^↑^ - PRL increase; ^↓^ - PRL decrease; ^↔^ - in contrast to*.

## Discussion

Despite the importance of this topic in clinical practice, an adequate substitute for Risperidone with a more favorable side effect profile has not been found. Since all APs have adverse effects, clinicians must choose the least harmful treatment strategies (64). A possible form of treatment for this side effect may be a replacement oral form for LAIR, but the finding in the current literature are contradictory (65, 66). In support of this, Bai et al. (65) demonstrated improvement in symptoms and reduction of prolactin levels in patients after LAIR. On the other hand, there are results without a positive association between prolactin reduction and LAIR (66).

General recommendations for Risperidone-related HPL include discontinuation or dose reduction, switching to a prolactin-sparing drug, adding dopamine agonists, or replacing estrogen in women. Several atypical APs with lower potential to block the D2 receptor have potentially more harmful long-term effects than Risperidone. The addition of dopamine agonists to reduce prolactin levels carries its risks and may worsen psychotic symptoms (67–71). Recent research suggests that aripiprazole (67), lurasidone, quetiapine (68), clozapine (69) may be used as prolactin-sparing drugs, and raloxifene and metformin (70) have also shown promise in terms of their efficacy in safely lowering prolactin levels (71).

Given that Risperidone acts predominantly on dopamine and serotonin receptors, which contain ion channels, it is justified to research whether potential ion substitution would prevent or mitigate the occurrence of these adverse events. A higher incidence of hypocalcemia has been shown on Risperidone therapy compared to other drugs. A significant correlation was found between the Risperidone dose and the calcium level and a significant and negative correlation of calcium level with study days (72). Milovanovic et al. (73) suggested that changes in serum calcium levels depend on the personality profile so that individuals who are susceptible to depletion and those prone to serum calcium accumulation have been identified.

Given the high efficacy of Risperidone in the treatment of many psychiatric conditions and the extent of the findings of HPL and prolactin-related symptoms, it is necessary to find an adequate way to use this drug with the least risk. Constant awareness of HPL and its adequacy can also have significant long-term health benefits. To improve the quality of life of these patients, clinicians must recognize and treat HPL associated with the use of these drugs. A “prolactin-sparing” AP would, therefore, significantly improve the patient's quality of life. In our opinion, further research should be directed in the direction of supplementation with certain minerals (especially calcium) (73). The presence of these ion channels and their supplementation in the lactotrophic cells of the pituitary gland may be the key to the safe use of the drug, minimal risk of side effects, as well as recovery from them.

## Author Contributions

MS conducted the search and data collection. BR, MJ, VJ, NM, and DR collaborated on the overall write-up with MS. All authors contributed to the article and approved the submitted version.

## Conflict of Interest

The authors declare that the research was conducted in the absence of any commercial or financial relationships that could be construed as a potential conflict of interest.

## Publisher's Note

All claims expressed in this article are solely those of the authors and do not necessarily represent those of their affiliated organizations, or those of the publisher, the editors and the reviewers. Any product that may be evaluated in this article, or claim that may be made by its manufacturer, is not guaranteed or endorsed by the publisher.

## Abbreviations

HPL: Hyperprolactinemia
AP: Antipsychotics
ASD: Autism spectrum disorder
LAIR: Long-acting intramuscular injection of Risperidone.
